# Building a Tiered Approach to *In Vitro* Predictive Toxicity Screening: A Focus on Assays with *In Vivo* Relevance

**DOI:** 10.2174/138620710790596736

**Published:** 2010-02

**Authors:** James M McKim

**Affiliations:** CeeTox Inc., 4717 Campus Dr., Kalamazoo, MI 49008, USA

**Keywords:** *In vitro*, predictive, toxicity, screening, hepatocytes, drugs.

## Abstract

One of the greatest challenges facing the pharmaceutical industry today is the failure of promising new drug candidates due to unanticipated adverse effects discovered during preclinical animal safety studies and clinical trials. Late stage attrition increases the time required to bring a new drug to market, inflates development costs, and represents a major source of inefficiency in the drug discovery/development process. It is generally recognized that early evaluation of new drug candidates is necessary to improve the process. Building *in vitro* data sets that can accurately predict adverse effects *in vivo* would allow compounds with high risk profiles to be deprioritized, while those that possess the requisite drug attributes and a lower risk profile are brought forward. *In vitro* cytotoxicity assays have been used for decades as a tool to understand hypotheses driven questions regarding mechanisms of toxicity. However, when used in a prospective manner, they have not been highly predictive of *in vivo* toxicity. Therefore, the issue may not be how to collect *in vitro* toxicity data, but rather how to translate *in vitro* toxicity data into meaningful *in vivo* effects. This review will focus on the development of an *in vitro* toxicity screening strategy that is based on a tiered approach to data collection combined with data interpretation.

## BACKGROUND AND RATIONALE FOR *IN VITRO* TOXICITY SCREENING

**Fig. (1):**
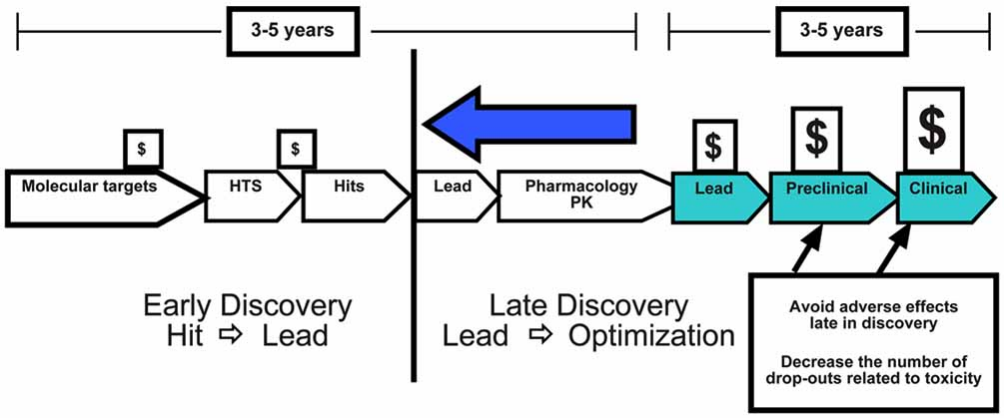
Diagrammatic representation of the major steps in the drug discovery process. A typical path for drug discovery is presented. Compounds in a chemical library are screened to identify those molecules that interact with the intended target. Molecules that are positive in this assay “Hits” begin the process of lead identification (Hit-to-Lead) and Lead optimization. *In Vitro* toxicity screening as well as screens designed to identify ADME, genotoxicity, and cardiac toxicity should be done early in this process in order to identify high risk molecules early.

**Fig. (2):**
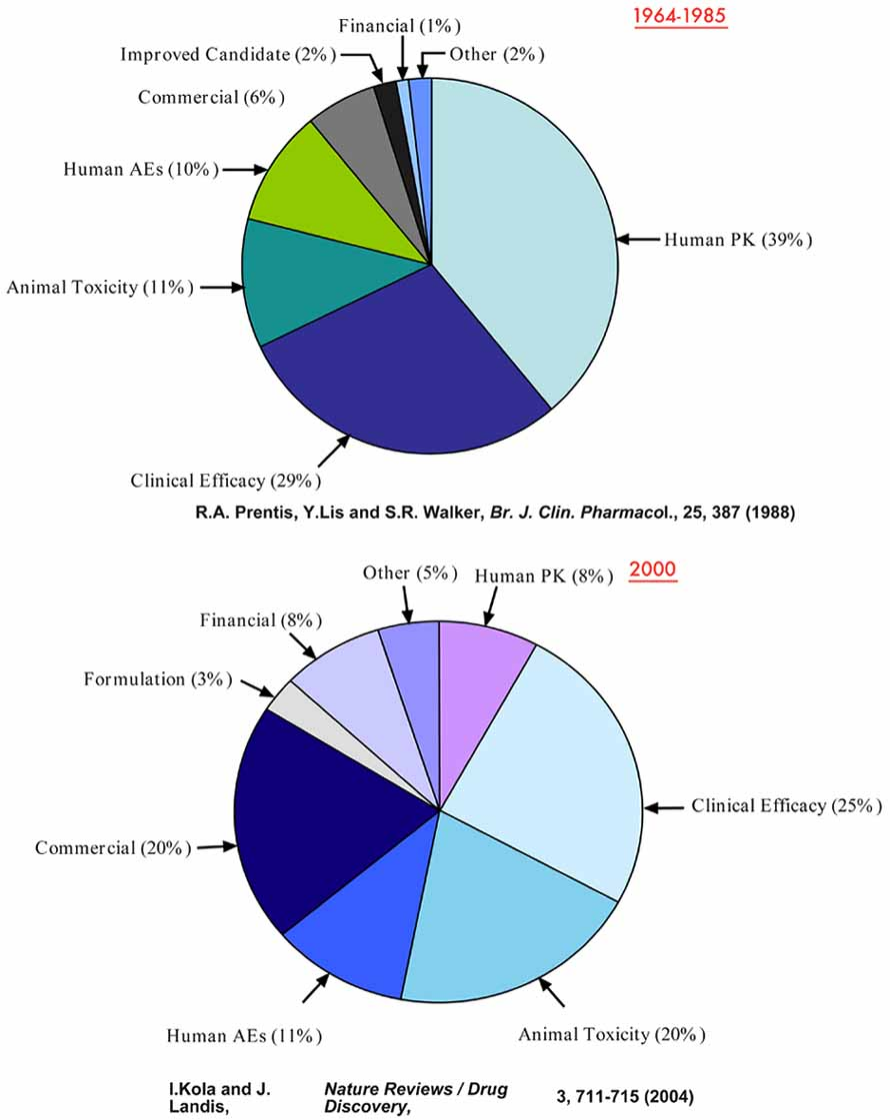
Reasons for drug failure in preclincial and clinical studies.

The discovery and development of a new pharmaceutical drug requires more than a billion dollars and can take 12 years of research effort. The drug development process can be divided into four distinct phases: early discovery, late discovery, preclinical, and clinical. When a new chemical with interesting biological properties enters this process, it will undergo extensive testing designed to address and solve many complex issues (Fig. **1**). New chemical entities or NCEs can be dropped from development for many reasons during this process, and the attrition rate for NCEs during preclinical and clinical studies is higher than it has been in decades. Although there are several factors that contribute to attrition, two major reasons are efficacy and toxicity (Fig.**2**). It has been estimated that only 1 of 10,000 chemicals that enter the discovery process ever reaches the market. This is not hard to imagine when one considers that 40% of NCEs that begin preclinical safety studies in animals will fail due to toxicity, and that 89% of NCEs that enter clinical trials will fail. The greatest cost in terms of time occurs in early and late discovery, which can require 6 to 8 years of research effort. In comparison, the highest cost in terms of dollars occurs in the preclinical and clinical studies. The ability to identify and reduce risk early can significantly improve the process of drug development by improving efficiency and improving the probability of success.

The high rate of attrition and the staggering cost of drug development mean that better decisions need to be made earlier. The evaluation of candidate drugs in animal safety studies should provide a means to verify safety, not identify toxicity. The new paradigm in drug discovery should include a robust means of identifying issues related to toxicity early in the discovery process where the cost of dropping a molecule is less than in later phases. The primary objective at this stage of development should be the simultaneous optimization of desired drug attributes, while at the same time identifying potential adverse effects. This approach would enable discovery scientists to select candidates for preclinical safety trials with the highest probability of success.

Many believe that the primary goal of the *in vitro* models for evaluating toxicity should be to predict human specific toxicity [1]. This may be due in part to the success that *in vitro* screening for certain absorption, distribution, metabolism, and elimination (ADME) endpoints has seen over the past 10 years. In 1991, the primary reasons for drug failure in clinical trials were related to pharmacokinetics and bioavailability. These factors accounted for 40% of the drugs dropped from development. *In vitro* systems designed to evaluate permeability, interaction with membrane transporter systems, and metabolic stability in cell models with human relevance reduced this failure rate to less than 10% by 2000 (Fig. **2**) [2]. The administration of one drug can significantly alter the plasma concentration, half-life, or toxicity of another drug. This Phenomenon is called a drug-drug interaction or DDI. Drugs that inhibit cytochrome P450 (CYP) enzymes, such as CYP3A4, or that can induce the production of CYP enzymes have a high probability of producing a DDI [3-5]. Because animals have significantly different CYP enzymes, gut physiology, and pharmacokinetics than humans, new drugs are typically not tested in animals under conditions that would reveal potential DDIs. Thus, in the case of permeability, bioavailability, metabolic stability, and potential DDIs, it makes sense to build *in vitro* screening models focused on predicting human outcomes.

The Food and Drug Administration (FDA) requires the submission of an Investigational New Drug (IND) package prior to manufacturing, transport across state lines, and testing in humans. A large part of the IND submission consists of animal safety studies. Typically, these studies are performed in a rodent (rat) and non-rodent species (dog).

Compounds that produce significant toxicity in animal models may never be tested in humans because they would never be allowed into clinical trials. An exception to this would be if the mechanism of toxicity in animals was unique to animals and thus not relevant to human safety. A classic example of this can be seen with phenobarbital and the associated liver enlargement, transient increases in liver enzymes in serum, and thyroid effects, which result in tumors in rodent carcinogenicity studies. Several studies have demonstrated that liver enlargement and development of tumors was due to phenobarbital induction of cytochrome P450 enzymes, as well as certain phase II conjugating enzymes that occur in rodents, but not in humans [6]. Thus, drugs and chemicals (e.g., loratadine and oxazapam) that display classical phenobarbital-like effects and produce tumors in rodents may still be approved by the FDA [7].

Until the FDA changes its process for evaluating the safety of new drug candidates, it will be important to incorporate cell-based models that can predict toxicity in rodents into early higher throughput screening paradigms. Then as potential candidates become visible *in vitro* models designed to predict human specific risk can be used. It is important to remember that a compound considered to be non-toxic in the rat may be toxic in another species including humans. One way to approach this challenge is to establish a multi-tiered *in vitro* screening approach designed to identify compounds that produce acute, chronic or delayed type toxicity in the rat, predict organ specific toxicity, identify species-specific toxicity, and determine potential risk factors for adverse effects in small patient populations which would include risk for idiosyncratic toxicity.

## BUILDING A TIERED TOXICITY SCREENING APPROACH

**Fig. (3):**
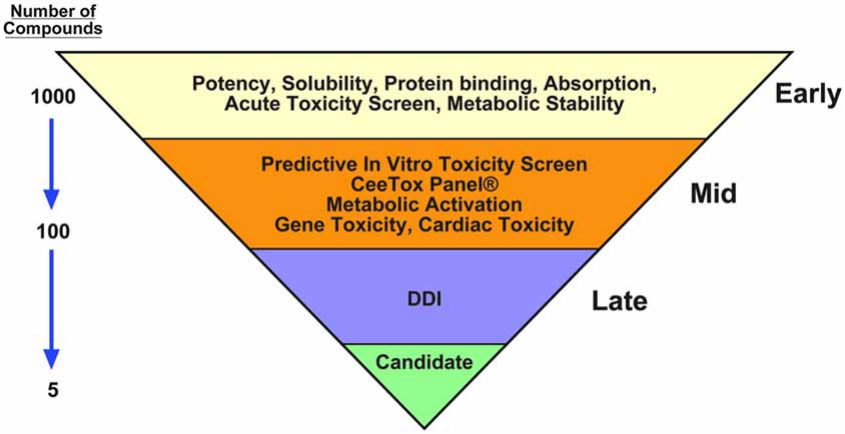
A tiered approach to early toxicity screening. A single *in vitro* screening platform is unlikely to provide the diverse set of data required to evaluate risk and predict *in vivo* toxicity. Therefore, a tiered systematic approach to *in vitro* screening should be employed. Solubility, protein binding, an understanding of the relationship between potency and toxicity, and the identification of severe toxicity should be an early consideration. This is followed by detailed information on the mechanism of toxicity and prediction of toxicity in rodents. The identification of compounds that would cause gene toxicity or have a high risk for producing cardiac toxicity should also be determined as early as possible. Potential issues related to drug-drug interactions (DDIs) and species-specific toxicity round out the toxicity profile in late discovery. Prior to selecting a candidate for preclinical development, the compound should be reviewed in terms of therapeutic area, risk/benefit scenarios, and anticipated duration of exposure.

The challenge is to incorporate methods for evaluating and understanding potential liabilities of NCEs into multiple phases of the drug discovery process. It is unlikely that any one *in vitro* model would be sufficient as a final decision point for toxicity, but rather a series of models that provide important information at the right time in the discovery pipeline should be used in a tiered approach (Fig. **3**). A well defined decision tree should also be part of this screening paradigm. In order to be useful, the *in vitro* toxicity screening models must be well characterized and predictive of *in vivo* effects with a low incidence of false positive or negative results. The system should have the capacity to test a large number of molecules in a short period of time with a minimum amount of compound. The data should provide information on potential mechanisms of toxicity, and subcellular targets. Early in the discovery process, when the number of molecules identified as “hits” can be large, *in vitro* cell-based toxicity assays can be used to rapidly assess potential safety issues associated with a new group of molecules. This information can be used in an iterative manner as chemists modify structures to improve drug-like properties, the modified compound can be retested for toxicity.

When the test compounds produce a half-maximal response in the cytotoxicity assays a toxicity index or TC50 can be determined. A typical early step in drug discovery is to optimize the potency of an NCE for the intended target, which is expressed as an IC50. When the toxicity (TC50) value is compared to the potency (IC50) value, an *in vitro* therapeutic index is obtained. This ratio can be used to determine the relative risk of toxicity between two or more molecules. As toxicities begin to emerge it is essential to know whether these are an extension of the intended pharmacology (a  target mediated effect) or whether they are due to off-target effects related to compound chemistry. If the toxicity of an NCE increases as potency is improved, the compound has less value because these data indicate that the undesired effects are the result of target interaction. If, on the other hand, there is a clear separation between the two parameters, the molecule holds greater promise. When the test compound produces severe toxicity *in vitro* and is anticipated to reach therapeutic plasma concentrations that are greater than or equal to the TC50 value the probability of *in vivo* toxicity is high.

NCEs that pass through this portion of the funnel begin late stage discovery, or lead optimization. Although the placement the utilization of specific assays and screens in the discovery process can vary, it is typically in this phase where potential drug candidates are screened for cardiac toxicity, gene toxicity, DDI potential, as well as risk for systemic acute and chronic toxicity. *In vitro* assays must have clear *in vivo* relevance so that discovery scientists have confidence that the effects observed *in vitro* accurately reflect *in vivo* events. There also must be a clear link between the *in vitro* toxicity data and the *in vivo* anchor, or reference, parameter. One example of an *in vivo* reference point is the plasma concentration attained at the lowest dose that produces toxicity in animals following repeated administration of the test compound. This parameter may also be used when extrapolating *in vitro* data to *in vivo* effects in humans. The ability to link *in vitro* results with *in vivo* plasma concentrations provides a solid reference point for assessing the relative risk for toxicity between several NCEs. *In vitro* assays that can predict general toxicity in rat 14-day repeat dose studies are important because they provide a means of evaluating *in vivo* risk relative to therapeutic area, potency, and estimated maximum therapeutic plasma concentrations. This information is invaluable when trying to discern from among a handful of potential candidates the one with the most desired drug-like properties and lowest risk profile.

Once a candidate and possibly a backup are selected for preclinical animal safety evaluations, it is important to identify and understand potential differences in toxicity between the test animals and humans. Early detection of potential species-specific toxicity issues prior to initiating an animal study can be provide valuable information that can be used to select a more appropriate test species. Moreover, when the mechanisms underlying species-specific toxicity can be elucidated, it is easier to provide perspective on what would be expected to occur in humans. The pharmacokinetics of a test compound can vary between species with clear effects on subcellular targets of toxicity, metabolism, the metabolites formed, and half-life. When the mechanism(s) of species-specific toxicity can be identified and characterized, these data can be used to argue that the toxicity observed in one test species is not relevant to human risk because the mechanism underlying the animal toxicity is not present in humans.

## SELECTING THE *IN VITRO* TEST SYSTEM

### Cell Lines

There are many cell and tissue models available for *in vitro* toxicity testing. A prudent approach is to understand both the strengths and weaknesses of each model. Early discovery screening requires a robust cell model that can be easily cultured for 24-72 h in 96 well culture plates. The cells should be genetically stable and provide reproducible results day-to-day. The cells should be well characterized in terms of their doubling time, optimal growth conditions, and biochemistry. There are many examples of cell lines that have been used to assess toxicity. These include, MDCK, Caco, V79, HUH7, HepG2, NRK-52E, 3T3, HEK293, and many more. When selecting a cell line as a model system in early screening, it is important to fully characterize and understand the morphology and biochemistry of the cell, including the species and organ from which it was derived.

One example of a useful cell model is the rat hepatoma (H4IIE) cell line which has several unique properties that make it a good choice for *in vitro* screening. These cells have been well characterized in terms of growth properties and their biochemistry. They were first introduced in the 1960s and have been well described in the literature. Their doubling time is approximately 22 h, and they can be cultured in a wide range of serum concentrations. The cells are sensitive to CYP1A inducers, such as 3-methylcholanthrene and dioxins, and as such have been used in bioassays to detect dioxin like molecules in environmental systems [8-11]. The cells possess a complete glutathione redox system [12, 13]. Glutathione S-transferase isoforms are present in high abundance [14]. The enzymes involved in the synthesis of GSH (glutamate-cysteine ligase and GSH synthetase) are also present and can be up regulated by exogenous chemicals [15]. Many cells in culture lose oxidative metabolism and convert to anaerobic metabolism (glycolysis) for energy production, a process known as the Crabtree effect. H4IIE cells are unique in that they maintain oxidative metabolism, a much more *in vivo* like property [16]. These cells also express high levels of P-glycoprotein and possess extremely low constitutive drug metabolizing activity. The low drug metabolizing activity means that the toxicity data obtained represents the effects of the parent compound.

### Primary Hepatocyte Cultures

Primary hepatocytes from rat, dog, monkey and human have been used extensively to evaluate chemical and drug toxicity, metabolism, bioactivation, transporter interaction, intrinsic clearance, and other biochemical processes [12, 17-20]. An important advantage of primary hepatocytes is that NCEs can be evaluated *in vitro* with cells prepared from normal tissue. This system also provides a convenient means of comparing compound toxicity in the same tissue type from different species including humans. Because primary hepatocytes have both phase I and phase II drug metabolizing systems they have been used extensively to study drug metabolism, and toxicity.

The use of primary hepatocytes for early higher throughput toxicity screening of NCEs is not practical due to the need for animals, time for isolation, variability between preparations  and cost. The routine use of human primary hepatocytes is even less desirable because of limited tissue availability, variation in isolation procedures, and inter-individual variation. The use of cryopreservation technologies for long term storage of primary hepatocytes provided a significant improvement to this model. Cryopreservation of human hepatocytes makes their use more feasible for routine screening. Variation between donors can be reduced by pooling hepatocytes isolated from multiple individuals. This cell system can provide valuable information on compound toxicity and metabolism if the source and quality of the cells are closely monitored. Day-to-day variation can be high, and the response and quality of the cells can vary greatly from one laboratory to another.

Primary hepatocytes grow optimally in the presence of serum protein. The cultures can be sensitive to both the serum concentration used and the source of the serum used. Optimal growth of hepatocytes is usually achieved with culture plates that have been coated with an extracellular matrix protein, such as Matrigel or collagen. Culture time is limited in most laboratories to a maximum of 72 h without significant loss of metabolic activity. Cytochrome P450 activity is typically inducible and present at constitutive levels with good metabolic capacity, provided the cells are of high quality and are cultured under conditions that optimize cell viability and maintain CYP activities.

Strict criterion for cell use should be implemented and adhered to in order to reduce variability between experiments. For example, cells that can not be established in monolayer culture may not be considered acceptable for use [12]. This cell model can provide important information related to species differences in metabolism and toxicity. It also provides information on CYP induction, an important parameter used to assess a compounds potential for causing DDIs. More specialized and complex culture techniques, such as sandwich-cultured hepatocytes can provide an *in vitro* model that is capable of identifying more specific types of hepatotoxicity, such as damage to bile cannilicular membranes known as hepatobiliary toxicity [21]. Human bile salt export pumps (BSEP) can be a target for some drugs and chemicals; therefore, inhibition of BSEP can result in hepatobiliary as well as generalized toxicity [22]. Bioactivation of a test compound occurs when metabolizing enzymes convert the parent molecule to a more toxic metabolite. Primary hepatocytes can be used to identify compounds that undergo metabolic activation and to determine how these metabolites affect cellular toxicity [23, 24].

### Precision Cut Tissue Slice

**Fig. (4):**
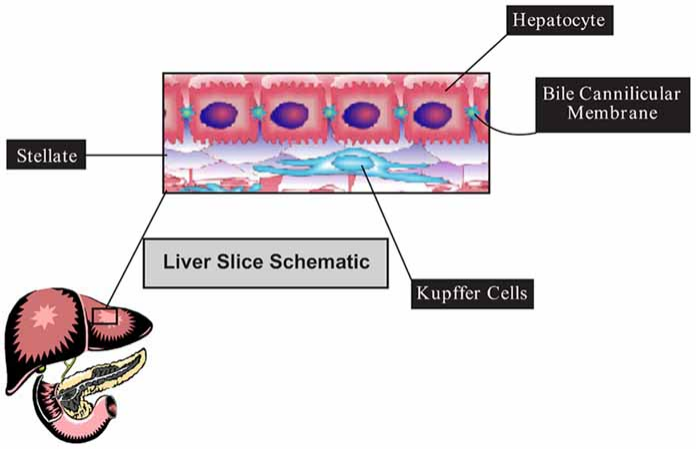
Diagrammatic representation of tissue architecture with precision cut tissue slice. The ability to assess whether a new drug candidate could produce hepatobiliary toxicity, or toxicity resulting from activation of Kupffer cells and subsequent release of chemical mediators, can only be tested in cell models that maintain a cellular architecture similar to the that *in vivo*. A key advantage of PCTS over primary hepatocytes and co-culture models is that the relative abundance of each cell type is maintained.

Precision cut tissue slice (PCTS) is a process whereby cores of excised tissue, such as liver can be sliced into discs of uniform thickness. The tissue slices obtained from this process can be incubated with test compounds and the effects on various biochemical functions monitored. This system has been used to investigate the toxicity of a wide range of drugs and chemicals. Both phase I and phase II metabolizing systems are intact [25] and inducible in PCTS [26]. Some advantages to this system include an intact organotypic architecture that is similar in composition to the original tissue. Most cell types present in the tissue are represented and each cell type can be found in the correct relative abundance. This allows evaluation of cell-to-cell interactions *in vitro*. For example, the effect of a test compound on one cell type (e.g., Kupffer) which may be stimulated to release mediators, such as cytokines that can then act on neighboring parenchymal cells to cause toxicity can be monitored [27-31] (Fig. **4**). Immunohistochemical and histological staining procedures can be used to evaluate the effects of compounds on cell health. Systems that require cell-to-cell communication, such as the hepatobiliary system, are also intact in the liver slice model. Thus, PCTS is an excellent model for evaluating drug of chemical induced hepatobiliary toxicity [32]. Multiple tissues from a single animal can be used to prepare slices, and this has been successfully done with liver, kidney, heart, and lung tissues [33-34]. Not only does this system provide a means of comparing compound effects across multiple organs, but it also allows for comparisons across multiple species. This means that the system can be applied to problems that require the ability to discern toxicity, including target organ toxicity, and species-specific toxicity [33-36].

One disadvantage of PCTS has been the relatively small number of slices that could be obtained from a single animal and tissue type making high volume screening less practical. However, recent studies by [37] describe a method of significantly increasing the number of slices by preparing small slices known as “chips”. The use of liver chips has the advantage of significantly increasing the number of test wells, and hence treatment groups, that can be analyzed. Another disadvantage that has limited the use of PCTS as an early screening tool has been the inability to cryopreserve slices, and to recover them with a high degree of viability. Recent efforts to improve cryopreservation techniques in different species have demonstrated that it is possible to cryopreserve tissue slices [38-40]. While most of the cryopreservation work reported has been with liver or kidney slices it will be necessary to develop and validate methods that can be applied to all tissues from which slices are made. Issues of viability related to time in culture, regional changes in viability due to the thickness of the slice, diffusion of nutrients, and the type of incubator employed for culture [41] must be tested and optimized for each laboratory. With continued improvements and optimization, PCTS can be an important tool in the arsenal available for evaluating the toxicity of drugs and chemicals *in vitro*.

### Important Physical and Chemical Properties *In Vitro*

*In vitro* cytotoxicity assays have been used by laboratories around the world for several decades. However, their ability to predict *in vivo* toxicity of unknown molecules has not been reliable. The term predictive implies that the toxicity results obtained *in vitro* can be related to a value *in vivo* that is indicative of dose limiting toxicity. One obvious example would be the use of *in vitro* data to estimate the plasma concentration in a rat 14-day repeat dose study where toxicity would be expected to occur. This value would then become the threshold for toxicity and would serve as a reference point for comparing molecules in a group.

There are several reasons why the *in vitro* cytotoxicity assays of the past have not provided a reliable means of predicting *in vivo* toxicity. When single endpoints are used to monitor cell viability, such as MTT or membrane leakage enzymes, there is a higher incidence of false positive and negative data. The use of one or two exposure concentrations instead of developing a full concentration response curve does not provide the kind of quantitative information required to extrapolate the *in vitro* effects to a relevant *in vivo* reference value, such as a plasma concentration, where toxicity would be expected to occur. In many instances the exposure concentrations used *in vitro* have little relevance to the maximum plasma concentrations achieved *in vivo*. Other important factors typically not addressed include protein binding, metabolic stability, metabolic activation, metabolites, temporal relationships, and compound solubility. In order for *in vitro* systems to predict accurately what will occur in animal or human systems, all of the parameters mentioned above, as well as others, must be addressed.

### Evaluation of Multiple Parameters Provides a More Accurate Toxicity Assessment

**Fig. (5):**
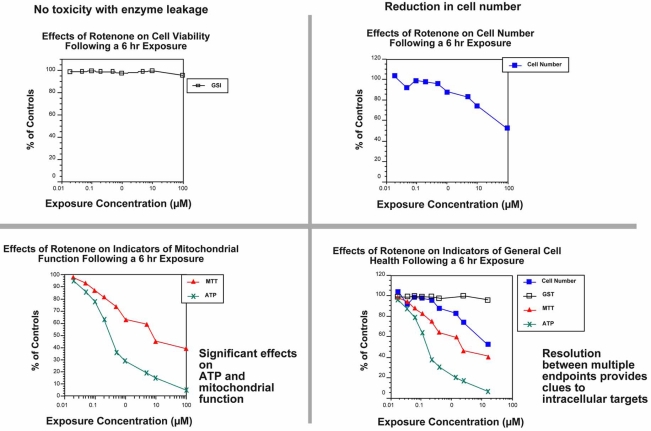
Importance of multiple endpoints, time and concentration-response curves. The toxicity of rotenone was evaluated with the rat hepatoma (H4IIE) cell line. Cells were seeded into a 96-well culture plate at a density of 10,000 cells per 100 µL of culture medium containing 20% serum. Following a 48 h equilibration period, rotenone was added at concentrations ranging from 0 to 100 µM and allowed to incubate at 37°C with 5% CO_2_ for 6 h. These data illustrate the importance of time, concentration-response, and multiple endpoint analysis for interpreting *in vitro* toxicity data. After 24 h, all of the biochemical endpoints respond in a similar manner, and resolution between response profiles is difficult. The addition of a shorter exposure time allows separation between the different endpoints. It is clear that mitochondrial markers (ATP and MTT) are most sensitive to rotenone. Each point on the graph represents a mean of 4-5 wells. The coefficient of variation ranged from 10-15%.

Drugs and chemicals can affect cell health at multiple levels at varying exposure concentrations and after different times of exposure. This means that certain subcellular targets such as mitochondrial function may be significantly affected before the cell dies and releases marker enzymes for viability into the media. This is an important concept when *in vitro* cell based biochemical or molecular data are intended to predict toxicity in either animals or humans. An example of this type of response profile occurs following a 6 h *in vitro* exposure to the pesticide rotenone, a known mitochondrial poison, at concentrations ranging from 0.1 to 100 µM (Fig. **5**).

The four panels represent commonly used cytotoxicity assays in many laboratories and include a membrane leakage enzyme (glutathione S-transferase, GST) in panel A, cell proliferation in panel B, and two markers of mitochondrial function MTT and ATP in panel C. Note that if membrane leakage (panel A) was the only marker used to assess toxicity, no effect would have been observed following a 6 h exposure, a false negative result. If cell mass or cell proliferation was the endpoint selected, there would have been a modest concentration dependent reduction, but it would not be clear whether this was due to cell death or an inhibition of cell proliferation. Regardless, the degree of toxicity would have been considered low, an incorrect conclusion. If the evaluation had been done by monitoring changes in ATP or MTT, a pronounced concentration response curve would have been observed (panel C). These endpoints would have provided a more accurate assessment of the toxic liability associated with rotenone; however, it would not be apparent from just these data whether the effects were due to cell death, changes in cell proliferation, or inhibition of the enzymes essential for assay performance. By combining several endpoints (panel D) it is possible to obtain a more complete toxicity profile of the test compound. In the case of rotenone, the mitochondrion is the most sensitive target, resulting in loss of ATP and reduced rates of cell proliferation prior to cell death, after a 6 h exposure. If the exposure time is extended to 24 h there is less resolution between the endpoints measured (Fig. **5**) [42, 43].

Redundancy in biochemical markers reduces false positive and false negative results. For example, the commonly used marker of cell health 3-[4,5-dimethylthiazol-2-yl]-2,5-diphenyl tetrazolium bromide (MTT) is reduced by metabolic enzymes (reductases) to a blue dye. The viability of the cell is directly proportional to the amount of blue dye produced. Test compounds that induce or inhibit these enzymes could produce erroneous results. Dicumarol is an example of a drug in this category [44]. It is also important to understand the limitations of each *in vitro* assay. MTT and Alamar Blue are considered to be markers of mitochondrial toxicity; however, the reductases involved with their conversion have been reported to be present in subcellular components outside the mitochondria [45]. This implies that changes in MTT or Alamar Blue readings may not be entirely due to changes in mitochondrial function. To improve interpretation of MTT and Alamar Blue data it is necessary to determine the role of mitochondrial reduction for each cell model. This can be done by monitoring MTT or Alamar Blue reduction by the test cells with and without antimycin A (10 µM), a known inhibitor of mitochondrial function. This method was used to demonstrate that the loss of Alamar Blue signal following drug exposure was primarily due to mitochondrial enzymes [46, 47] in neuronal cells. Knowing the true contribution of mitochondrial reductases to changes in MTT and Alamar Blue can dramatically improve the interpretation, and hence, the predictive power of the *in vitro* data.

### The Importance of Reference Points and Multiple Parameters

The idea that cells in culture, separated from other cell systems and endogenous control mechanisms, can predict the toxicity of an unknown substance is like sitting in a boat in the middle of the ocean on a cloudy night with no visual reference points from which to navigate. Simply growing cells, adding test compound and measuring a biochemical response does not provide a reference point from which to predict *in vivo* toxicity. In order to interpret *in vitro* data in a manner that will provide the highest predictive power, it is necessary to establish reference points both *in vitro* and *in vivo*. These reference points can then be used to predict *in vivo* toxicity. *In vitro* reference points with the highest value are solubility, metabolic stability, cell death (membrane integrity), cell number, solubility, and logP. Some key reference points *in vivo* include maximum plasma concentration, solubility, protein binding constants, and bioavailability.

**Fig. (6):**
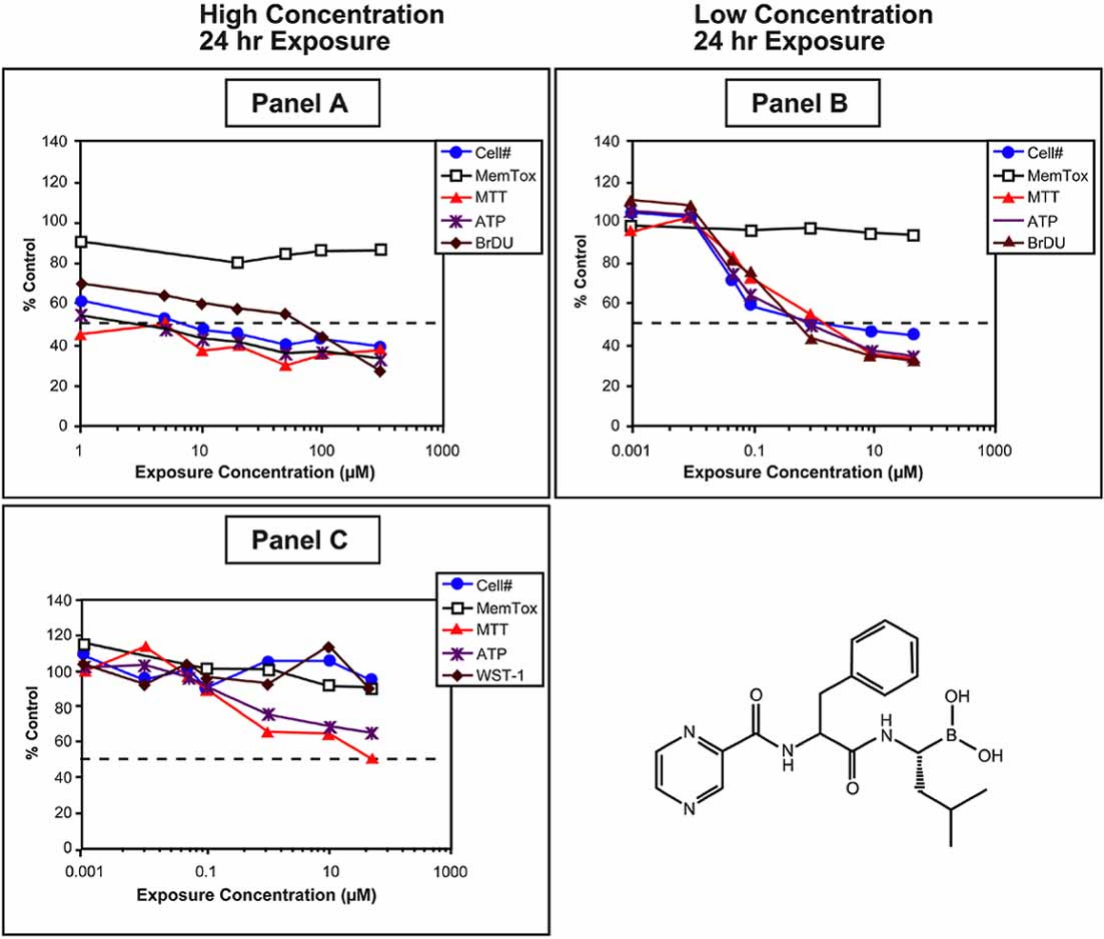
*In vitro* biochemical toxicity profiles that identify cytostatic drugs. Velcade is a first in class proteasome inhibitor prescribed for the treatment of cancer. The drug provides a good example of the importance of reference points for the interpretation of *in vitro* data. The drug was tested in the rat hepatoma (H4IIE) cell line as described under Fig. (**5**). In panel **A**, the biochemical cell based markers (ATP, MTT, and cell proliferation) appear maximally affected at the lowest exposure concentration. Note that the marker for cell viability (GST leakage) is essentially unaffected. *In vitro* data should be normalized to cell number, thus the fact that all cell based endpoints follow the loss of cell number indicates a cytostatic effect. In order to confirm this interpretation, exposure concentrations are reduced in order to define the full response curve. In panel **B**, a classical exposure-response curve was produced. Again, all cell-based markers are dependent on cell number. This means that the only true effect on the cells under the conditions tested was on cell proliferation, which is a desirable effect for an anticancer drug. If a single marker, such as ATP or MTT had been used, the interpretation would have been that the compound was toxic. If a membrane leakage marker had been used, the conclusion would have been that the drug had no toxicity. When cytostatic drugs are identified by *in vitro* tests, it is important to evaluate the drug in a normal non-proliferating cell model. In panel **C**, velcade was evaluated in rat primary hepatocytes. There was no change in cell viability or cell number, but there was a reduction in ATP and MTT indicating effects on mitochondrial function.

*In vitro* evaluation of the oncology drug velcade (bortezomib) demonstrates how important multiple parametric analysis is for the correct interpretation of *in vitro* data (Fig. **6A**). This drug is a proteosome inhibitor that was approved for the treatment of cancer by the FDA [48, 49]. The drug inhibits cell proliferation and causes cell death by caspase dependent and independent processes [50]. The data shown in Fig. (**6A**, **B**) represent the biochemical changes observed following a 24 h exposure to concentrations of velcade ranging from .001 to 300 µM in the rat hepatoma (H4IIE) cells. Following the exposure period, all of the cell based assays for cell health (ATP, MTT, Cell mass) are maximally affected at the lowest exposure concentration with a maximum response of approximately 50% (panel **A**). In comparison, the membrane leakage marker for cell viability (glutathione S-transferse (GST)) was essentially unchanged. This response profile is a general finger print for cytostatic drugs or chemicals.

The H4IIE cells double approximately every 22 h and in this example the exposure was for 22-24 h. Because of this, a test compound that inhibits cell proliferation could not achieve more than a 50% response. Therefore, the data in Fig. (**6A**, **B**) indicate that the velcade potently inhibited cell proliferation as determined by both the incorporation of 2’-bromodeoxyuridine (BrdU), and cell mass at the lowest exposure concentration tested. The other cell based assays responded in a similar manner, but in this case the loss of ATP and MTT was not directly related to mitochondrial toxicity or to cell death, but rather to a reduction in cell number. When this profile is observed during the *in vitro* testing of compounds it is important to establish the full concentration response curve. By lowering the exposure concentration range of velcade, the entire response profile could be defined (Fig. **6B**). Cell viability remained high as indicated by the lack of response seen with the membrane leakage enzyme GST. It should be evident from this example that cell based endpoints, such as ATP depletion or MTT reductase activity, without reference points, such as cell proliferation and cell death, are considerably more difficult to correctly interpret.

When a test compound has its’ primary effect on cell proliferation, and it is being tested in a proliferating cell line, it is important to evaluate the molecule in a normal non-proliferating cell system to more completely characterize potential undesired effects from responses that may be desired (Fig. **6C**). Note that in normal rat primary hepatocytes, which do not undergo extensive cell division, there was no effect on cell mass, but there was a concentration dependent reduction in ATP and MTT, two markers of mitochondrial health. In the case of velcade, inhibition of cell proliferation without cell death would be a desired response for an oncology drug. Inhibition of cell proliferation per se might not be considered a cytotoxic effect. However, if normal cell populations that undergo high rates of cell division *in vivo*, such as bone marrow cells, were inhibited for a prolonged period, the effects would be considered adverse.

Another example of the importance of multiple endpoint analysis for correct interpretation of *in vitro* toxicity data can be seen with the drug methotrexate, which was intended to be used as an antiproliferative for the treatment of cancer. The *in vitro* cytotoxicity data shown in Fig. (**7**) demonstrate that the drug potently inhibits cell proliferation to a maximum of 50% without acute cell death. The drug also effectively induces apoptosis as determined by caspase 3 activation. An undesired side effect of this drug is mitochondrial damage and oxidative stress leading to liver toxicity during therapy [51-53]. In Fig. (**7**) there is a potent concentration-dependent loss in cellular ATP that is independent of the reduction in cell number. Although methotrexate is an effective antiproliferative drug used to treat cancer it may also cause undesired toxicity. Multi-parametric *in vitro* assays can provide important clues about potential undesired effects.

The relative sensitivity of various biochemical endpoints can provide important insights into potential subcellular targets and mechanisms of toxicity [43]. In addition, the use of multiple parameters of cell health can greatly improve the interpretation of *in vitro* cytotoxicity data and reduce the incidence of false positive or false negative results. Endpoints should provide information on both acute type toxicity and delayed or more chronic type toxicity. Some types of toxicity require time to develop [54]. Acute markers such as membrane integrity, mitochondrial function, and cell proliferation must be balanced with chronic indicators of toxicity, such as glutathione depletion and lipid-peroxidation.

**Fig. (8):**
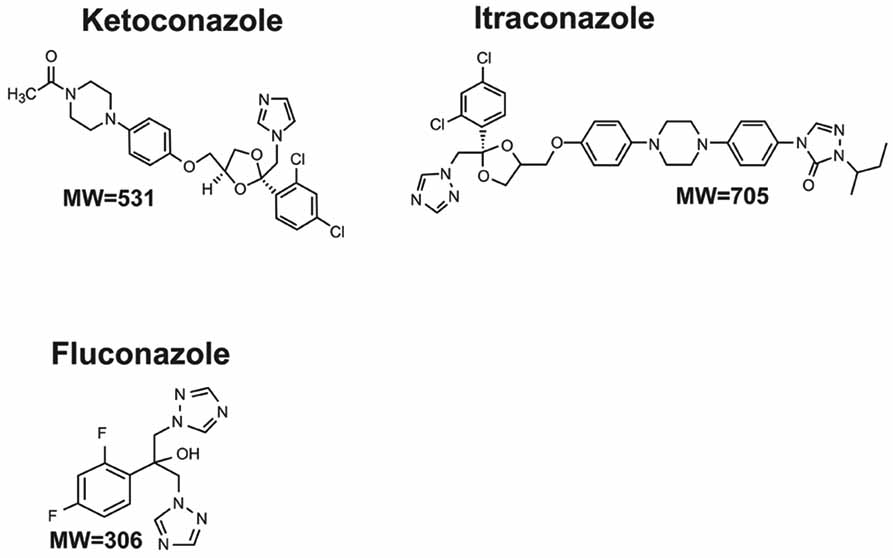
Chemical structures of three antifungal drugs.

In another example, *in vitro* multi-parametric analysis was used to evaluate the relative toxicity of three antifungal drugs: ketoconazole, itraconazole, and fluconazole. Fluconazole has fewer structural similarities than ketoconazole and itraconazole (Fig.**8**). Although these drugs were developed and marketed at different times, it is useful to analyze the compounds as if they had been three new NCEs in late stage discovery and only two could be selected and brought forward into animal safety studies. In 1995 two of the molecules, ketoconazole and fluconazole, were evaluated in an *in vitro* primary hepatocyte system [55]. These experiments showed that ketoconazole was considerably more cytotoxic than fluconazole at therapeutically relevant exposure concentrations. Moreover, it was reported that one mechanism of ketoconazole toxicity was linked to mitochondrial toxicity [56]. The toxicity profiles shown in Fig. (**9**) compare all three antifungal drugs in the H4IIE cell model combined with concentration response, and multi-parametric analysis. Ketoconazole caused mitochondrial toxicity and reduced the rate of cell proliferation. Ketoconazole was the most cytotoxic, with fluconazole having the safest profile. The estimated blood concentration where toxicity would first be expected to occur (Ctox) is also shown in Fig. (**9**). The predicted toxicity value of 55 µM obtained from the *in vitro* model was in close agreement with actual rat and human pharmacokinetic and toxicity data. Remembering that the toxicity data should only be one piece of the information used to select candidates for development, it is useful to review the potency/efficacy data for the three drugs. For the endpoints of potency/efficacy selected, all three drugs were similar. An evaluation of ADME data relevant to toxicity such as CYP inhibition indicates that ketoconazole and itraconazole are potent inhibitors of CYP3A4, the enzyme responsible for a large portion of drug metabolism in humans. This means that co-administration of drugs that are metabolized primarily by CYP3A4 (simvastatin, lovastatin, terfenadine, midazolam) with ketoconazole or itraconazole can result in clinically significant drug-drug interactions (DDIs) [57]. In comparison, fluconazole is a weak inhibitor of CYP3A4 and would therefore have a lower risk for DDIs when co-administered with drugs metabolized by CYP3A4. However, this compound is a potent inhibitor of CYP2C9 and CYP2C19 [57, 58] and therefore drugs that are primarily metabolized by 2C9 (phenytoin, warfarin, sulfamethoxazole, losartan) and co-administered with fluconazole could also produce DDIs. If all of these drugs had been developed in the same project, the best molecules for continued development would have been itraconazole and fluconazole as they carry less risk of toxicity produced by the parent drug and lower potential for DDIs. Although there is reason for concern regarding potential DDIs with the antifungal compounds discussed here, this risk must be weighed against the risk of not having an effective treatment for systemic fungal infections.

In early drug discovery, *in vitro* evaluations of new drug candidates is often met with skepticism because of a fear that a compound might be “killed” by a false positive result before it has an opportunity to become a drug. Although there is some validity to this argument, it is important to put *in vitro* data into context relative to other important pieces of data. A compound that tests with a high degree of toxicity must be evaluated relative to other desired drug attributes. The investigators must ask whether the toxicity is related to potency and hence target mediated or whether the toxicity is unique to the chemistry. Is the pharmacophore of the molecule the same as the toxicophore? The therapeutic area of the compound and risk to benefit rationale must be applied. Will the drug be administered for two weeks or for the life time of the patient? Is the drug intended to treat a life threatening disease or a headache? What is the estimated maximum therapeutic plasma concentration during the course of treatment? A matrix of this type of information can be prepared that will provide a more realistic picture of the risk profile for a new drug candidate.

**Fig. (10):**
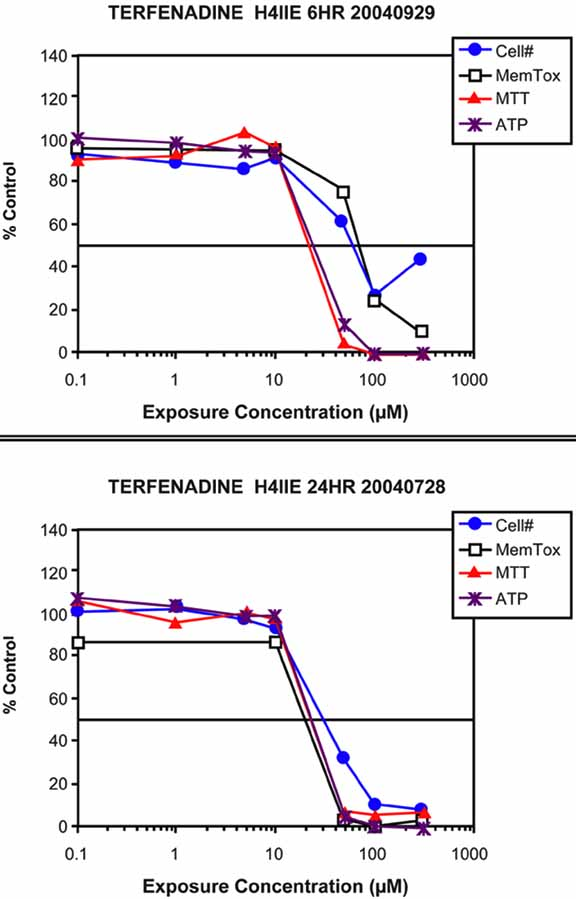
*In vitro* toxicity profile for terfenadine, an example of a false positive. Terfenadine produced significant toxicity at both 6 and 24 h. If this compound had been evaluated in an early discovery program, the in vitro data would have indicated toxicity. This compound was successful in all IND enabling studies. The reason for the low levels of toxicity measured *in vivo* is first-pass metabolism. Terfenadine has a bioavailability of <1% and undergoes first-pass metabolism. Thus, *in vivo* plasma concentrations of parent drug are below toxic levels. *In vitro*, where metabolism is low, parent compound can reach concentrations that can produce toxicity.

In order for *in vitro* toxicity data to be used in the decision making process, it must be complete, mechanism-based, and reliable with a low incidence of false positive and false negative results. Again, *in vitro* and *in vivo* reference points can greatly improve the predictive power. Terfenadine was marketed as seldane, an antihistamine that would not cause drowsiness. In January of 1997 the FDA proposed removing terfenadine from the market because of potentially fatal cardiac effects observed in patients who were also receiving other commonly prescribed medications, including antifungal agents in the azole class, such as ketoconazole. The cardiac toxicity was the result of a drug-drug interaction with compounds that have been shown to be potent mechanism-based inhibitors of cytochrome P450 (CYP) metabolizing enzymes (reviewed by [59]). When terfenadine was evaluated in an *in vitro* model over an exposure concentration range of 0 to 300 µM for 6 and 24 h, significant toxicity was observed (Fig. **10**). These data indicate that if terfenadine had been evaluated in early discovery with an *in vitro* toxicity model, it might have been identified as having substantial toxicity. However, this drug made it to market; it passed the preclinical safety studies and clinical trials, but caused significant cytotoxicity *in vitro*, a false positive result.

Understanding why this compound showed less toxicity *in vivo* than is key to improving the *in vitro* toxicity screening process. Terfenadine undergoes first-pass metabolism [60], with a reported bioavailability of less than 1%. The primary metabolite fexofenadine is not only efficacious, but less toxic [61, 62]. The extremely low plasma concentration of terfenadine translates to low toxicity. Unlike the *in vivo* situation most cells (including the H4IIE cells) in culture have low metabolic capacity. Thus, in the *in vitro* system the parent drug terfenadine remained high and toxicity related to ion channel disruption was observed (Fig. **10**).

**Fig. (11):**
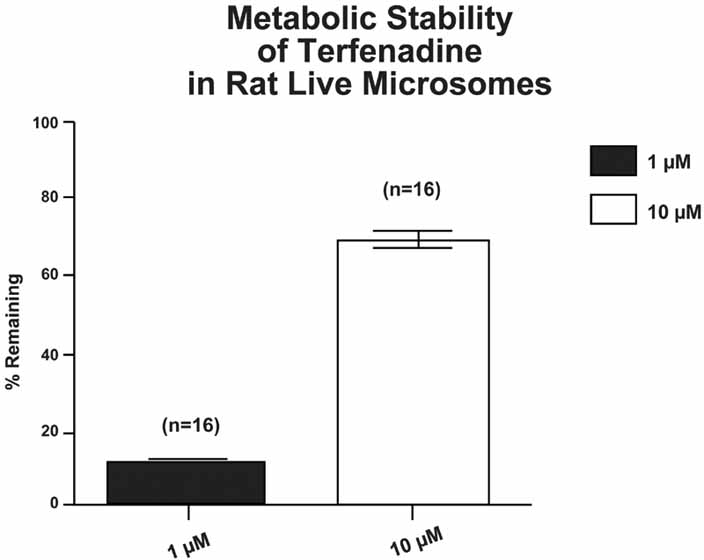
Metabolic stability of terfenadine (Seldane). Rat microsomes were used to evaluate the metabolic stability of terfenadine (Seldane). Microsomes were diluted to a final concentration of 0.5 mg/mL in phosphate buffered saline (PBS), pH 7.3, with NADPH (100 µM), and test drug at 1.0 and 10 µM. Following an incubation of 30 min, the amount of parent drug remaining was determined by using LC-MS. The data are expressed as the percent of parent remaining (%R). Seldane and midazolam are examples of drugs that have low metabolic stability. They are subject to first-pass metabolism and typically have low bioavailability. *In vitro* systems with low metabolic capacity evaluate the cytotoxic effects of parent drug. In order to reduce misinterpretation of these data, it is important to determine metabolic stability.

It is important to know whether a new chemical entity tested *in vitro* is metabolically stable. One way to test this is to use microsomes to determine the percent of parent remaining after a 30 min incubation of 1 and 10 µM drug (Fig. **11**). Liver microsomes are a convenient way to compare the metabolism of a test compound between species. Compounds that cause toxicity *in vitro*, but have low metabolic stability, would be flagged as potential false positives. Compounds that have low toxicity *in vitro*, and undergo extensive metabolism *in vitro*, may produce significant toxicity *in vivo* due to formation of reactive metabolites. The significance of this in relation to terfenadine is even more poignant when one considers that although the parent molecule was withdrawn from the market, its primary metabolite fexofenadine is currently sold as Allegra^®^.

### Protein Binding and Cytotoxicity

**Fig. (12):**
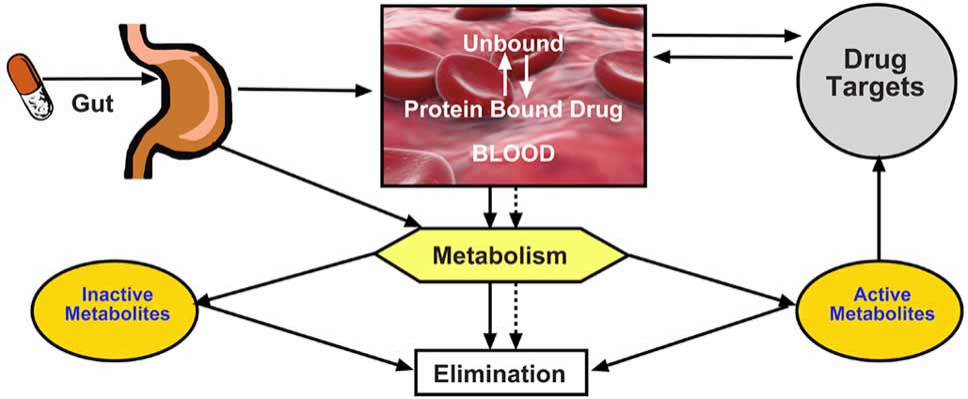
The Interaction of a drug with plasma protein is dynamic.

The efficacy, toxicity, and half-life of a drug can be influenced by the amount and type of its association with plasma proteins. Drugs in biological systems exist in two forms, protein bound and free. The primary classes of proteins with which drugs can be associated are albumin, lipoprotein, glycoprotein, and globulins. It is the free portion that is available to cells and tissues, and which elicits desired or undesired effects. The affinity of a drug for protein determines availability. Compounds with low affinity will rapidly dissociate from protein to reestablish equilibrium. Thus the interaction of most drugs with plasma proteins is a dynamic and reversible process, with bound-drug moving to the free-drug so rapidly that it is considered, for all practical purposes, to be fully available to tissues (Fig. **12**). This means that the rate of dissociation from protein is not rate limiting for organ or cell exposure when equilibrium can be rapidly established. This is due to the time required for a drug to move from protein-bound to free (milliseconds) versus the time required to diffuse into cells (seconds) [63].

The presence of serum protein in the cell culture system is an important parameter to consider when extrapolating *in vitro* toxicity data to *in vivo* effects for two primary reasons: 1) cell health and 2) cellular exposure to drug. Obviously, under *in vivo* conditions serum protein is 100%. Typically, *in vitro* systems include 10% fetal bovine serum (FBS) in the culture medium. Many cell lines and primary cultures are sensitive to the serum concentration and their viability is dependent not only on the correct concentration used, but also on the source of the serum. Serum should be purchased from reputable sources that have a quality control program to ensure consistency batch-to- batch. Once a satisfactory lot of serum has been identified it is a good practice to purchase it in quantity and store frozen in order to maintain the consistency of the vitro data. The rat hepatoma cell line (H4IIE) can be cultured in serum as low as 2.5% and as high as 20%. In general the presence of serum protein is beneficial as it not only creates an optimal growth environment for the cells, but it can facilitate the solubility and hence the cellular exposure of difficult compounds. It is important to understand protein binding kinetics of potential drug candidates. The ability of a molecule to dissociate (Kd) from the protein determines whether protein binding will limit exposure. Test compounds with a high percent association with protein and a low binding affinity (high Kd) readily dissociate and in this case the protein will most likely improve solubility and delivery to the cells. In contrast, compounds with a high percent association with protein and a high binding affinity (low Kd) will be much slower to reestablish equilibrium, between free and bound drug and protein binding will have a greater influence on cellular uptake (Fig. **12**).

**Fig. (13):**
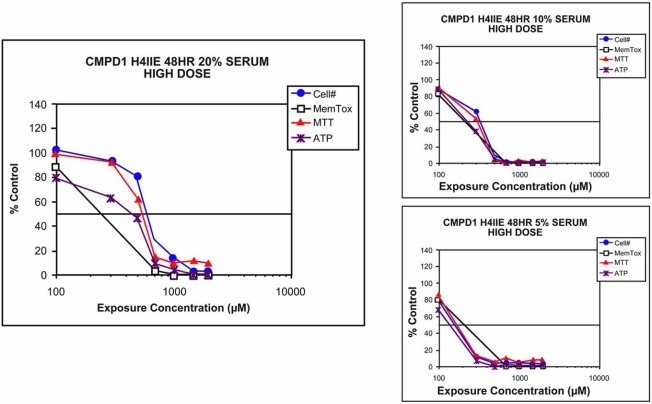
Effects of protein binding on `*in vitro*` toxicity. The toxicity of drugs with a high affinity for proteins (low Kd) is affected by the amount of protein in the in vitro test system. The response profiles depicted in the graphs demonstrate how the toxicity of a drug can be changed by the amount of protein in the test system. Compounds evaluated early in discovery that show low *in vitro* toxicity in the presence of high protein concentrations (>10%) should be tested for protein binding. In the example shown, the test compound was evaluated in the presence of 20, 10, and 5% serum. Values represent the mean of 4-5 wells. The coefficient of variation was between 10% and 15% across the assays. Standard error of the mean bars are not shown for clarity.

New drug candidates with high affinity protein binding are less desirable. *In vitro* screening data that are negative for toxicity should be a flag for potential protein binding. The effect of protein binding on cytotoxicity can be addressed by conducting a serum titration experiment. The test drug is exposed to the cell system in the presence of decreasing amounts of serum protein (Fig. **13**). A shift in the response curve to the left indicates high affinity (low Kd) binding. The data for the test compound shown in Fig. (**13**) indicate how the toxicity of a drug that has high binding affinity for plasma proteins is controlled by the amount of protein present in the *in vitro* system.

### Importance of Exposure Time *In Vitro*

The length of exposure time *in vitro* can have dramatic effects on the toxicity profile for a compound. The goal should be to determine an *in vivo* time point to which the *in vitro* data can be modeled. A standard early toxicity evaluation in rodents is the 14-day repeat dose study most often carried out in rats. If this model is used as the *in vivo* reference for *in vitro* toxicity evaluations, then the *in vitro* data should provide a reasonable prediction of general toxicity (liver, kidney, bone marrow, heart) based on an *in vivo* target or reference point, such as maximum plasma concentration at the lowest dose where toxicity was first observed. The challenge then is to determine the exposure time *in vitro* that would enable a prediction of plasma concentration *in vivo* where toxicity occurs. It should also be understood that increasing exposure times *in vitro* can have two effects; one is that the curve does not shift in terms of the IC50, but the maximum effect achieved at higher exposure concentrations increases, and the other is that the entire response curve shifts to the left. Increased time usually shifts the toxicity response profile to the left *in vitro*; however in most cases there is a limit to this shift. For example, the greatest increase in toxicity (shift to the left) occurs between 6 and 24 h and between 24 and 48 h with little or no shift between 48 and 72 h. This phenomenon was reported by [64] in which sodium arsenite was evaluated for toxicity in a human osteogenic sarcoma (U-2OS) cell line. Concentration response curves were developed following 24, 48, and 72 h of exposure. The greatest increase in toxicity was observed between 24 and 48 h with only small changes between 48 and 72 h.

An *in vitro* screening program should provide data to discovery teams in a time frame of two to three weeks. Thus, it is advantageous to establish the *in vitro* model using the shortest exposure time possible. In theory, one could select any time as long as the algorithm for predicting the *in vivo* plasma concentration after 14-days of repeated exposure corrects for *in vitro* exposure time. Exposure times greater than 24 h are logistically difficult in an early screening paradigm. Exposures less than 24 h may not allow enough time for toxicity to occur, a situation that could produce a large number of false negative results. Each laboratory must develop a series of *in vitro* to *in vivo* comparison studies that enables the time correction to be determined for the exposure time selected.

There are instances when shortening or extending the exposure time can provide important information regarding the mechanism of toxicity or help explain why negative effects at 24 h may indicate delayed toxicity, which is not observed in a 14-day study, but does appear in 28 or 90-day animal studies.

### Design and Interpretation of Retrospective Studies

Ideally, new *in vitro* models for predicting *in vivo* toxicity of potential drug candidates should be validated in a prospective manner. An unknown molecule is screened in the *in vitro* system, to develop the predictive value, and then the molecule is evaluated *in vivo* to determine the accuracy of the *in vitro* data. In reality, this scenario is almost never the case because of the enormous expense and time required to generate the amount of data necessary to establish the validity of an *in vitro* system.

An alternative approach is to test large numbers of approved drugs retrospectively in a blinded manner. It is important that these studies are designed in a way that reduces bias in the experiment. Drugs from multiple therapeutic areas should be selected and if possible this should be done by a third party and not by the testing laboratory. Testing drugs that are known to cause hepatotoxicity and then demonstrating cytotoxicity in a liver cell does not confirm the predictive value of the *in vitro* assay. There must be a link to an *in vivo* parameter, such as plasma concentration, in order to consider the results of such a study valuable in terms of predicting *in vivo* toxicity. The *in vitro* data should provide a means by which to make a statement about the expected toxicity  *in vivo*. The *in vitro* data must stand alone and should provide a way to estimate what will occur *in vivo*. The question then is not whether the *in vitro* assay correctly identifies a compound already known to be toxic, but rather its ability to predict that an unknown molecule will be toxic at relevant plasma concentrations. The cell model selected may not allow the detection of specific types of toxicity where the mechanism is organ specific. A liver cell model may not identify a cardio-toxic compound if the mechanism of toxicity is unique to heart tissue. The ability to characterize risk for adverse events in animals, as well as humans, provides a way forward for early discovery teams who are faced with making go-no-go decisions during lead optimization. The primary objective is to select a candidate with the highest probability of success in preclinical and clinical safety studies.

The results of a large and comprehensive retrospective study were recently reported by [54]. The overall objective of this study was to “test the hypothesis that clinical occurrence of human hepatotoxicity concorded with *in vitro* cytotoxicity assessed in a cell-based model with a novel combination of critical features and using HCS.” In this study 243 approved drugs were selected based on their reported clinical hepatotoxicity and placed into four categories: 1) severe hepatoxicity, 2) moderate hepatotoxicity, 3) non-toxic, and 4) toxic to organs other than liver. The toxicity of each drug was measured in an *in vitro* high content screening (HCS) system to determine if the *in vitro* toxicity assays could detect and differentiate between drugs known to cause differing degrees of hepatotoxicity in humans.

A human hepatoma cell line (HepG2) was used as the test system in this study. Multi-parametric data were collected to assess cell health, including calcium, mitochondrial membrane potential, DNA content, cell number, and plasma membrane permeability. These data were collected over several exposure concentrations. The exposure time was based on the time that provided the most complete concentration response profile and was set at 3 days (72 h). This study successfully demonstrated the value of multiple endpoints, the varying sensitivity of biochemical endpoints, and the need for concentration response, and time optimization. These conclusions were in agreement with those reported previously [42, 43]. A component missing from the O’Brien [54] study, is the assessment of multiple concentration response curves to develop a single value that can be used to predict *in vivo* responses. The authors do assign relative risk to each test compound by developing a toxic index (TI). The TI is the ratio of the lowest cytotoxic concentration to Cmax and was based on the most sensitive endpoint responding at the lowest exposure concentration, not on a weighted analysis of all endpoints. This assumes that the most sensitive endpoint is the most significant in terms of predicting hepatotoxicity.

The study does provide a means of assessing the potential risk of human hepatotoxicity associated with one drug relative to others in the same class. However, the ability to discern relative risk between molecules must be based on a combination of important information sets. The *in vitro* toxicity profiles evaluated against or with therapeutic plasma concentrations, length of exposure, and the therapeutic risk/benefit value of the drug. The values selected for Cmax may not be the maximum plasma concentrations achieved during therapy in the clinic or the sources used to obtain Cmax data may be in error. For example, azathioprine is used as an immunosuppressant to manage inflammatory disease processes. The compound is known to produce myelotoxicity and hepatotoxicity at therapeutic concentrations [65]. The maximum therapeutic plasma concentrations reached during therapy is approximately 5-7 µM, a much different exposure scenario than the 0.34 µM Cmax value reported and used to calculate the TI [54].

**Fig. (14):**
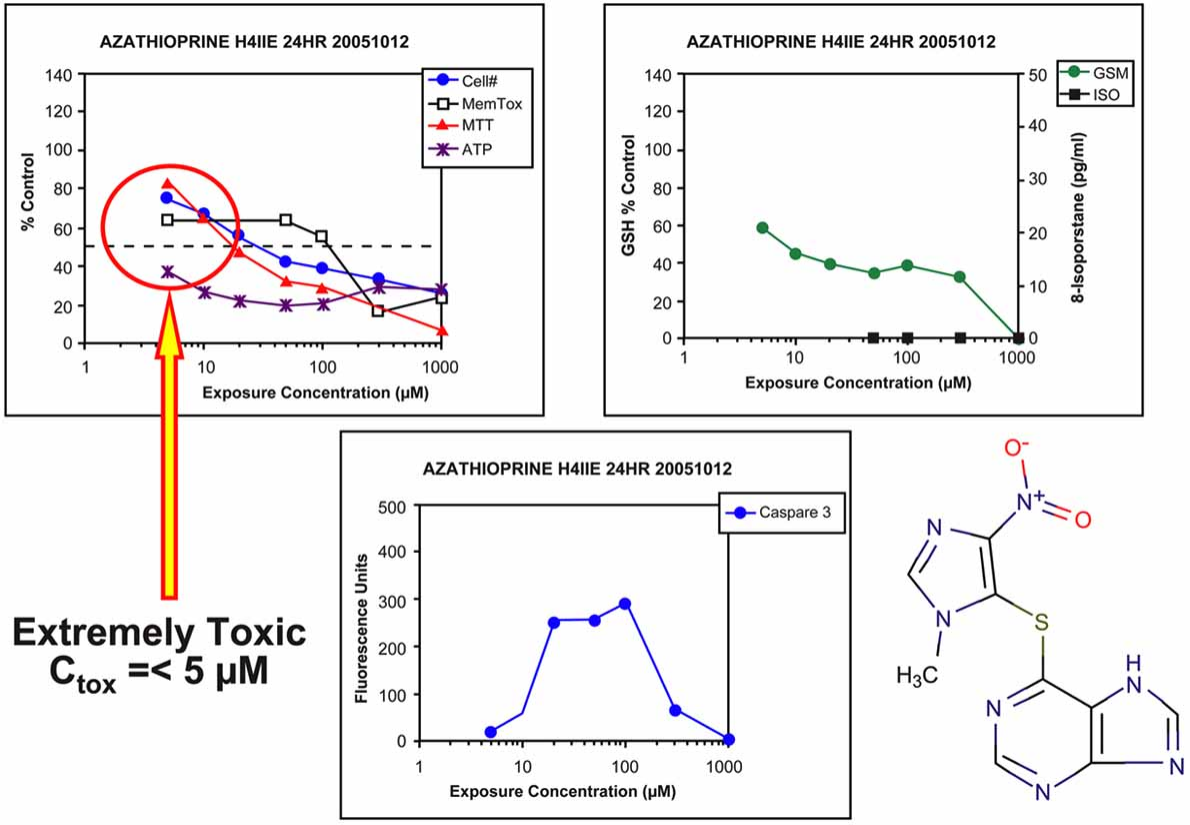
*In vitro* cytotoxicity of azathioprine. Rat hepatoma (H4IIE) cells were exposed to azathioprine as described under Fig. (**5**). The most sensitive subcellular endpoint was depletion of ATP and this is consistent with reports from other laboratories. This compound is highly toxic and is known to cause toxicity in patients during therapy. Values represent the mean of 4-5 wells. The coefficient of variation was 10-15% across the assays. Standard error of the mean bars were omitted for clarity.

The HCS method for assessing the hepatotoxicity of drugs was compared to a panel of standard cell based cytotoxicity assays performed independently, such as membrane integrity, DNA synthesis, protein synthesis, glutathione depletion, superoxide secretion, and caspase-3 activation. When azathioprine was evaluated no toxicity was detected in the standard cell-based assays [54]. These findings are inconsistent with those reported by several other laboratories where significant mitochondrial toxicity and depletion of cellular ATP following *in vitro* exposure of various hepatic cell lines to azathioprine at exposure concentrations and time similar to those reported [54] (Fig.**14**) [65-69].

Rotenone is a well characterized mitochondrial poison that causes pronounced toxicity in cells [43]. Although the toxicity of rotenone was identified by HCS in the O’Brien [54] study, the compound was not evaluated in conventional or standard cytotoxicity assays in this study [54]. Finally, the ability to resolve risk between drugs with similar TI values, but with different risk profiles, such as paroxetine TI=4, acetaminophen TI=4, and cerevastatin TI=3 also requires further consideration. Thus, there does not appear to be a clear advantage between the HCS method and the use of standard cell based assays that monitor toxicity. The point here is that multiple endpoints can and do improve the predictive power of the *in vitro* system and that the method or platform used to collect data is not nearly as important as the process used to interpret the meaning of the data.

The predictive power of *in vitro* screening is improved with the inclusion of endpoints associated with chronic toxicity. If these endpoints are affected in a concentration - dependent manner by drugs that have been withdrawn from the market due to unanticipated adverse events, but not by drugs in the same class considered to be safe, the information can provide risk profiles for predicting this toxicity.

An analysis of several drugs removed from the market because of hepatotoxicity revealed that at least three key parameters were affected: ATP, GSH, and apoptosis. One example of this can be seen with a group of antibacterial drugs known as the fluoroquinolines. Two members of this group (grepafloxacin and trovafloxacin) were withdrawn from the market due to unanticipated toxicity in some patients. The acute toxicity markers (membrane integrity, mitochondrial function, and cell proliferation) for these two drugs showed only a small reduction. All of the drugs in class would have been predicted to be safe based on the *in vitro* toxicity data and acute markers. By evaluating markers associated with more chronic toxicity, it was shown that the two drugs withdrawn caused small reductions in ATP, and a pronounced loss in cellular GSH levels. The changes in biochemical function were supported by changes in gene expression profiles [70]. This relationship between ATP, GSH, and apoptosis with idiosyncratic liver toxicity has also been demonstrated with other drug groups, such as the glitazones, and statins where some members have been shown to produce liver toxicity in a small number of patients.

In order for *in vitro* biochemical readouts of toxicity to be predictive of *in vivo* effects, many parameters must be optimized. In our laboratory the predictive power of the *in vitro* data is greatly improved by evaluating metabolic activation, metabolic stability, and CYP induction. Simply collecting *in vitro* cytotoxicity data is not sufficient for careful evaluation of risk around promising new drug candidates. There are also different levels of predictive *in vitro* toxicity evaluations. In early discovery, emphasis is placed on providing data that help predict whether a compound will be toxic in rats during 14-, 28-, or 90-day repeat dose safety studies. Ideally, an estimate of the blood concentration where dose limiting toxicity would first be expected to occur is most useful. This information can help scientists understand how toxicity and potency are related and provide a risk profile for a group of molecules in the hit-to-lead and lead optimization phase of drug discovery. This information can also provide a means of estimating an *in vitro* margin of safety. A margin of safety and a risk/benefit assessment can be determined by comparing the estimated plasma concentration where toxicity would be expected to occur, to the maximum therapeutic concentration anticipated during a course of therapy.

**Fig. (15):**
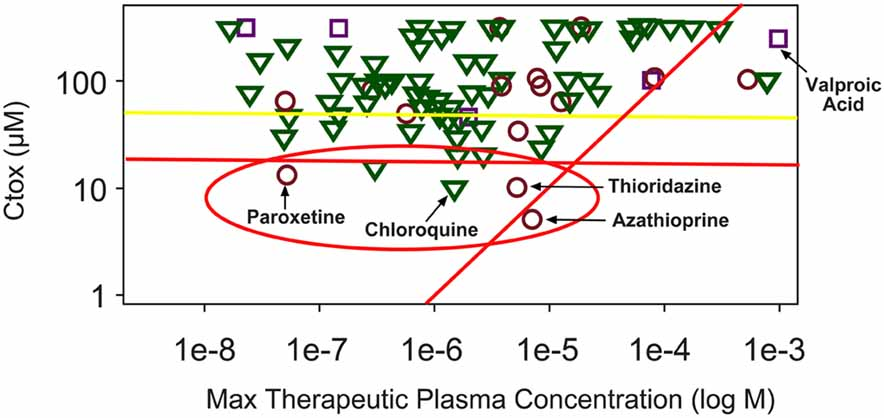
*In vitro* toxicity screening data combined with clinical data show good concordance. In this experiment, 150 approved drugs were selected and analyzed in a blinded manner. The aim of the study was to determine the relationship between the estimated plasma concentration where toxicity would be expected to occur in rat 14-d repeat dose studies (Ctox), and the maximum therapeutic plasma concentration (MTPC) achieved during the course of therapy in humans. The diagonal red line represents the threshold of toxicity where the estimated plasma concentration for toxicity is equal to the maximum plasma concentration measured in humans. If the Ctox value has any relationship to MTPC, most approved drugs should not exceed this threshold value. The data above indicate that 97% of the approved drugs do not achieve plasma concentrations equal to or greater than the predicted level of toxicity. If the drugs tested had been screened for toxicity early in their discovery life-cycle, those that had estimated plasma concentrations for toxicity that fell below 20 µM would have been flagged as toxic. The horizontal red line depicts this point on the graph. Most of the approved drugs are above this line, however some fall below, and therefore might be considered false positives in the *in vitro* screen. Upon closer inspection, these compounds all have been associated with toxicity in human patients. The key to why these drugs are still used in the clinic is related to their potency, *in vitro* margin of safety, and risk/benefit analysis. It is clear that as drug plasma concentrations approach the threshold value (Ctox) of toxicity the probability of an adverse event increases. This graph was developed by Dr. Georgor Zlokarnik and the work was part of a collaborative research project between CeeTox, Inc. and Vertex Pharmaceuticals.

In the study depicted in Fig. (**15**), 150 approved drugs were selected by a third party and presented in a blinded manner for evaluation in a panel of biochemical assays (CeeTox Panel^®^). The intent of this study was to compare an estimated plasma concentration (C_tox_) where toxicity would be expected to occur in rat 14-day repaeat dose studies, derived from the *in vitro* data, to the maximum therapeutic plasma concentration (MTPC) achieved during the course of therapy in the clinic. The diagonal red line depicts the point where the predicted toxicity value (Ctox) is equal to the MTPC. Most approved drugs should not reach plasma concentrations that are greater than this predicted threshold for toxicity. Clearly, most of the drugs (97%) fall to the left of this line. Those compounds that have MTPCs that meet or exceed this line of equivalence are in some cases drugs that are known to cause toxicity under therapeutic conditions, such as azathioprine. What about false positives? If the drugs in Fig. (**15**) had been evaluated in an *in vitro* toxicity screening program early in the drug discovery process those with predicted toxicity values (C_tox_) of 20 µM or less would have been considered to have a high probability of causing toxicity in animal studies. As expected the majority of approved drugs have estimated toxicity values greater than 20 µM. However, some would have been identified as toxic by the *in vitro* system (Fig. **15**, red circle). Unmasking some of these molecules reveals that an important factor to be considered for continued development of NCEs lies in the intended use of the drug (risk/benefit), its potency, and, margin of safety.

Paroxetine is a highly prescribed drug with relatively few incidences of toxicity in the clinic. This compound did produce cytotoxicity *in vitro* with a predicted Ctox value of 15 µM. The primary reason for the high degree of safety in the clinic is due in part to the high potency and hence lower MTPC (0.3 µM) reached during treatment. By comparing the estimated plasma concentration for toxicity (Ctox) to the MTPC an *in vitro* margin of safety can be developed. The data in Fig. (**15**) indicate that as a compound’s MTPC approaches the predicted toxicity value (diagonal red line) the probability of toxicity increases and the margin of safety decreases.

It should also be noted that most of the drugs tested have MTPC values between 0.1 and 10 µM. This indicates that *in vitro* exposure concentrations that range from 0.1 to 300 µM cover a range of concentrations that are therapeutically relevant. These data indicate that the predicted threshold for toxicity *in vivo*, determined using the CeeTox Panel, does provide information about human risk.

Discovering that a new drug candidate has no significant toxicity in rats, but causes mortality in dogs is a costly finding when discovered during preclinical animal studies. In most instances species-specific toxicity is related to pharmacokinetic or pharmacodynamic differences. Species-specific toxicity can be addressed with *in vitro* systems that allow direct species comparisons, such as primary hepatocytes and liver microsomes from rat, dog, and human. Metabolic capability and metabolite profiles are important parameters. If a metabolite is responsible for the observed toxicity in one species and that metabolite is not formed in the species resistant to toxicity the mechanism underlying the species-specific toxicity can be resolved.

In conclusion, the evaluation of adverse effects and the development of toxicity profiles around new chemical entities can greatly improve the selection process of new drug candidates. This in turn should reduce compound risk and increase the probability of success during preclinical animal studies and clinical trials. Ideally, early screening programs should begin during the hit-to-lead phase and continue through candidate selection. A decision tree that incorporates physical chemical, ADME, potency, protein binding, and toxicity should be developed and adhered to during the discovery process. Multiple endpoint analysis, concentration response curves, temporal relationships, metabolic stability and metabolic activation are essential to building a robust screening program. The platform used to collect the data is not as important as the quality of the data and the process used to interpret the data. *In vitro* toxicity data should be linked to an *in vivo* parameter that can be easily measured and is closely associated with toxicity. *In vitro* toxicity data should not be viewed in isolation, but rather combined with other key parameters and characteristics of a successful drug in order to make the most informed selection of new drug candidates.

## Figures and Tables

**Fig. (7):**
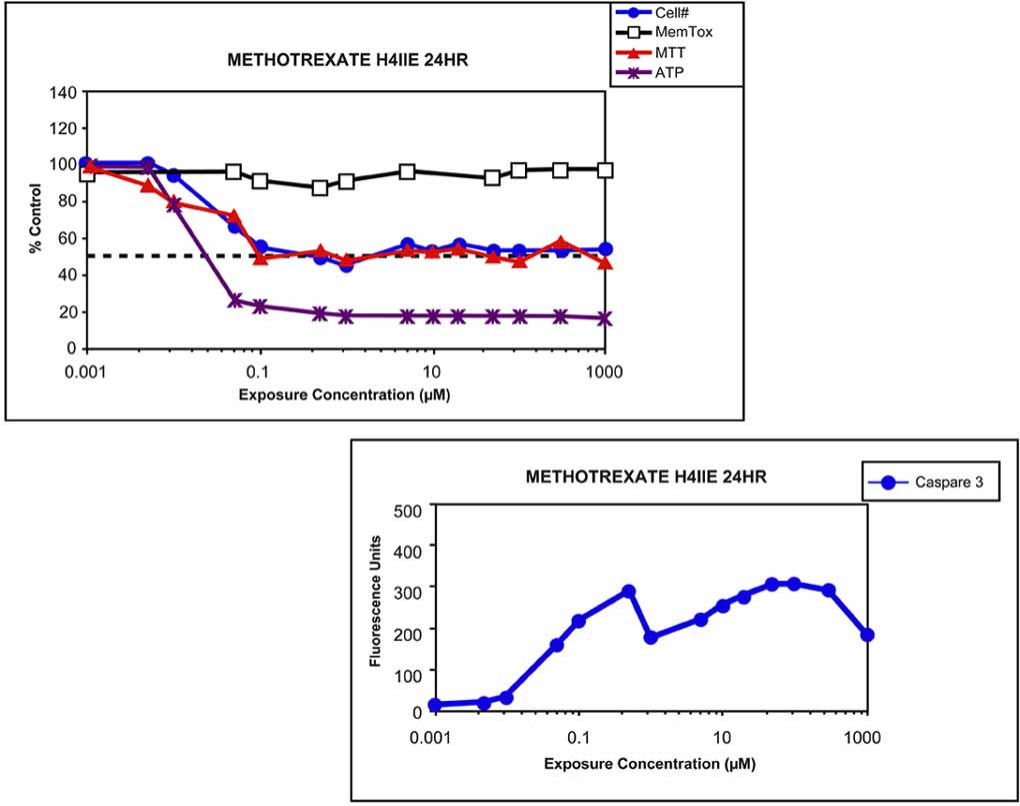
*In vitro* toxicity profiles produced by methotrexate. Rat hepatoma (H4IIE) cells were used as the test system under the conditions described under Fig. (**5**). Concentration response data, measured with several markers of cell health, show a pronounced reduction in cell proliferation. A 50% response is a maximum response given that the exposure time was 24 h and the doubling time for these cells is 22 h. Cell viability remains high in the face of inhibited cell proliferation. MTT is dependent on cell number; however, the observed reduction in cellular ATP was independent of cell number. If MTT had been the only assay used to assess toxicity, the compound would have appeared toxic. If membrane leakage had been used to measure viability, the compound would have shown low toxicity. By combining several key endpoints that measure cell health, it is possible to determine the primary effect, most sensitive subcellular target, and intended effect versus unintended toxicity.

**Fig. (9):**
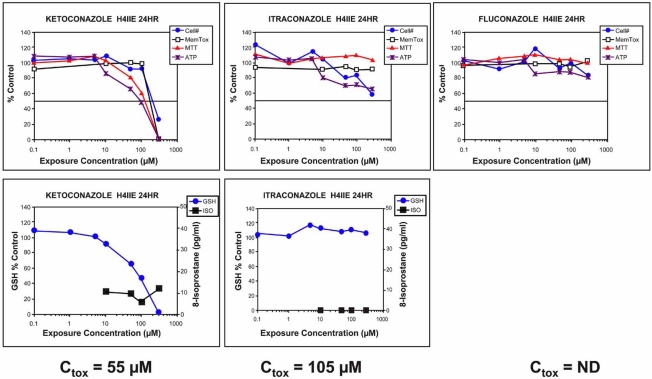
*In vitro* toxicity screening differentiates toxicity between drugs in the same class. The antifungal drugs ketoconazole, itraconazole, and fluconazole ll have a similar mode of action but were not developed at the same time. If the three molecules had been part of a single discovery program, *in vitro* toxicity screening could have provided important information regarding the relative safety of these drugs. To demonstrate this, all three drugs were evaluated in the rat hepatoma (H4IIE) cell line according to conditions described under Fig. (**5**). Under the conditions tested, ketoconazole showed the highest potential to produce toxicity, while fluconazole showed the lowest potential to produce toxicity. This interpretation is consistent with clinical observations. Values represent the mean of 4-5 wells. The coefficient of variation was between 10 and 15% across the assays. Standard error of the mean bars are not shown for clarity. C_tox_ = estimated plasma concentration at steady state where toxicity would be expected to occur in liver, kidney, bone marrow, or heart. ND = not determined.
